# Matrix stiffness‐induced α‐tubulin acetylation is required for skin fibrosis formation through activation of Yes‐associated protein

**DOI:** 10.1002/mco2.319

**Published:** 2023-07-12

**Authors:** Dongsheng Wen, Ya Gao, Yangdan Liu, Chiakang Ho, Jiaming Sun, Lu Huang, Yuxin Liu, Qingfeng Li, Yifan Zhang

**Affiliations:** ^1^ Department of Plastic and Reconstructive Surgery, Shanghai Ninth People's Hospital Shanghai Jiao Tong University School of Medicine Shanghai China

**Keywords:** acetylation, biomechanics, skin fibrosis, YAP, α‐tubulin

## Abstract

Skin fibrosis, a pathological process featured by fibroblast activation and extracellular matrix (ECM) deposition, makes a significant contribution to morbidity. Studies have identified biomechanics as the central element in the complex network of fibrogenesis that drives the profibrotic feedback loop. In this study, we found that the acetylation of α‐tubulin at lysine 40 (K40) was augmented in fibrotic skin tissues. Further analysis showed that α‐tubulin acetylation is required for fibroblast activation, including contraction, migration, and ECM deposition. More importantly, we revealed that biomechanics‐induced upregulation of K40 acetylation promotes fibrosis by mediating mechanosensitive Yes‐associated protein S127 dephosphorylation and its cytoplasm nucleus shuttle. Furthermore, we demonstrated that the knockdown of α‐tubulin acetyltransferase 1 could rescue the K40 acetylation upregulation caused by increased matrix rigidity and ameliorate skin fibrosis both in vivo and in vitro. Herein, we highlight the critical role of α‐tubulin acetylation in matrix stiffness‐induced skin fibrosis and clarify a possible molecular mechanism. Our research suggests α‐tubulin acetylation as a potential target for drug design and therapeutic intervention.

## Graphical abstract

1. Matrix stiffness-induced α‐tubulin acetylation is required for skin fibrosis formation.
2. Acetylated α‐tubulin at K40 promoted fibroblast activation by dephosphorylating Yes‐associated protein and maintaining its nuclear retention.
3. Downregulating K40 acetylation of α‐tubulin through knockdown of α‐tubulin acetyltransferase 1 could ameliorate fibrosis formation.

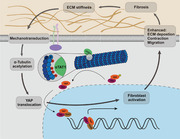

## 1 INTRODUCTION

Clinically, a series of skin fibroproliferative diseases including hypertrophic scarring (HS), keloid, and scleroderma demonstrate one common characterization, that is skin fibrosis.^1^, ^2^ Patients with skin fibrosis experience itching, restricted movement, and pain that decrease their quality of life. Issues with their physical appearance can delay their reintegration into society. Many approaches exist to minimize fibrosis progressions, such as corticosteroid injection, laser therapy, cryotherapy, and surgical removal.^3^ However, there is no satisfactory prevention or intervention option.

Skin fibrosis features dermal fibroblast activation and excessive extracellular matrix (ECM) deposition.^4^ The ECM is mainly synthesized by myofibroblasts, the activated type of fibroblasts with fibrogenic and contractile properties, and serves as a scaffold for cell movement and vascularization.^5^, ^6^ The main components of the ECM are fibrous macromolecular proteins, such as procollagen and elastin.^7^ Perturbation of collagen production and degradation is the hallmark of fibrosis. The overexpressed collagens are aligned in a single direction parallel to the epidermis, while collagens in normal skin are arranged in a nonparallel manner.^8^ The ECM in fibrotic tissue contains less elastin than that in normal skin, contributing to the lack of elasticity.^9^ The orientation and composition of the ECM are the main causes responsible for the change in the mechanical properties of scar tissue. As determined by atomic force microscopy (AFM), fibrotic skin tissues show an enormous increase in Young's modulus, which is reflected by the increase in substrate stiffness.^10^, ^11^

The increased matrix stiffness provides a stronger mechanical tension force inside the microenvironment, and mechanical tension drives the initiation and development of fibrosis.^12^, ^13^ Cells, especially fibroblasts, are highly sensitive to mechanical cues.^14^, ^15^ The stiffened and high‐tension ECM could influence cellular processes and plays an important role in the transduction of profibrotic signals. The three‐dimensional shape of cells and cell behaviors, such as migration, contraction, and proliferation, are also regulated by the actin cytoskeleton and microtubules when exposed to mechanical pressure.^16^ The cytoskeleton system is the bridge transmitting mechanical signals through the cytoplasm until the nucleus to control gene expression. The mechanical dynamics of microtubules are central to cell physiology. Among the crucial factors that mediate the functions of the microtubule network, posttranslational modifications (PTMs) of tubulin play a significant role. Detyrosination, acetylation, and phosphorylation of tubulin are the most studied PTMs of the microtubule cytoskeleton.^17^ In an in vivo model of tubulin directly subjected to fluid shear stress, intraluminal acetylation was found to be upregulated and increased flexibility and resistance to breakage.^18^ Responding to substrate rigidity sensing, cells acetylated microtubules to promote mechanosensitive cellular responses such as migration and contraction by promoting the fusion of post‐Golgi carriers at focal adhesions (FAs) and increasing FA turnover.^19^ The bioactivity of transcriptional coactivator Yes‐associated protein (YAP) has also been tightly linked to the actomyosin cytoskeleton. The contractile F‐actin structure, stimulated by stiff ECM and integrins, was observed to sustain YAP nuclear localization and activity.^20^ Collectively, despite emerging as a major regulator of mechanotransduction, the contribution of microtubules in scar progression remains elusive.

In this study, we proved that the acetylation of α‐tubulin at lysine 40 (K40) was augmented in fibrotic skin tissues, and biomechanics‐induced upregulation of K40 acetylation could induce fibrosis by inducing the YAP cytoplasm‐nucleus shuttle. Furthermore, we applied an adeno‐associated virus (AAV) to knock down α‐tubulin acetyltransferase 1 (αTAT1), the enzyme that specifically acetylates K40 of α‐tubulin, in a bleomycin‐induced skin fibrosis model and found less fibrosis formation with downregulated K40 acetylation levels.

## 2 RESULTS

### 2.1 K40 acetylation of α‐tubulin is increased in human fibrotic skin tissues

**FIGURE 1:**
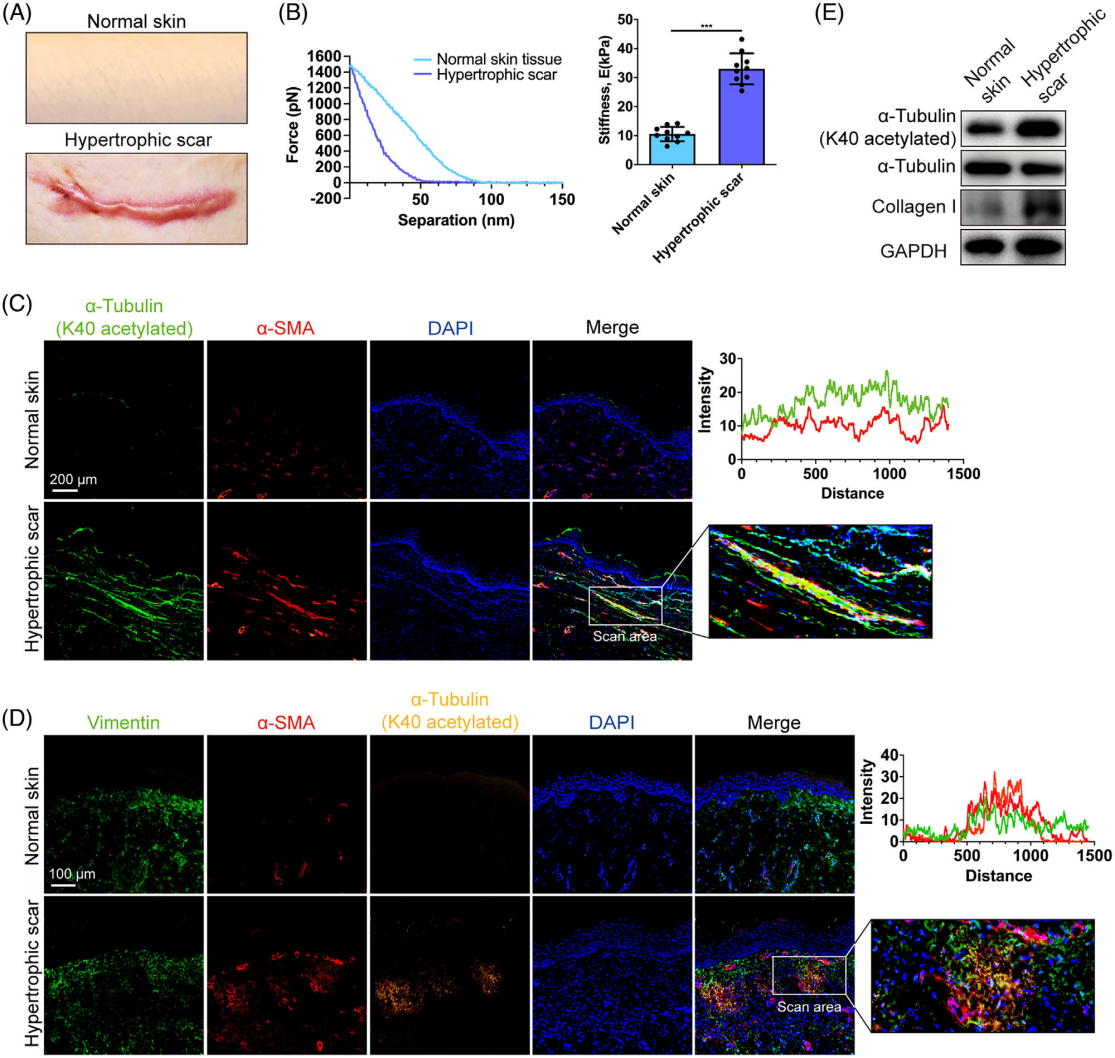
Expression of K40 acetylated α‐tubulin in normal and fibrotic human skin tissues. (A) Images of normal human skin and HS human skin. (B) Atomic force microscopy (AFM) measurements and quantitative analysis of normal and HS human skin tissues. Statistical tests: *t*‐test. (C) Images and colocalization analysis of immunofluorescent staining for α‐tubulin (K40 acetylated) (green) and α‐SMA (red) in normal and HS tissues (scale bar = 200 μm). An area where α‐SMA(+) fibroblasts were highly expressed was chosen for colocalization scanning. (D) Images and colocalization analysis of immunofluorescent staining for α‐tubulin (K40 acetylated) (yellow), α‐SMA (red), and Vimentin (green) in normal and HS tissues (scale bar = 100 μm). An area where α‐SMA(+) fibroblasts were highly expressed was chosen for colocalization scanning. (E) Western blot assay of α‐tubulin (K40 acetylated) total α‐tubulin and collagen I in normal and HS tissues. Data are presented as the mean ± SD (n = 10 biologically independent human samples). ***p < 0.001.

As one of the most common types of skin fibrotic disorders, HS (Figure 1A) is a thickened, elevated scar that develops within the wounded area and often lacks the flexibility of normal skin and thus can restrict movement. We investigated the mechanical properties of HS and normal skin using AFM. The Young's modulus of HS was significantly higher than that of normal skin (Figure 1B), indicating the high tension and stiffness of HS. As α‐tubulin supports the cell and directly responds to mechanical stress, we then analyzed the expression of K40 acetylated α‐tubulin, one of the most important PTMs of α‐tubulins,^21^ in human HS tissues and normal skin. Immunofluorescence analysis showed that the expression of K40 acetylated α‐tubulin was notably higher in HS, and the colocalization analysis revealed that the expression of K40 acetylated α‐tubulin was correlated with alpha Smooth Muscle Actin (α‐SMA) in HS tissues, suggesting that K40 acetylated α‐tubulin is mainly expressed in the α‐SMA(+) myofibroblasts in human HS tissues (Figure 1C, D). Western blotting showed a significantly increased expression of collagen I in HS tissues than in normal skin, which confirmed the fibrotic property of HS tissue (Figure 1E). Meanwhile, we also validated the upregulation of acetylated α‐tubulin at K40 in HS tissues (Figure 1E). In general, human fibrotic HS tissues presented an augmented acetylation level of α‐tubulin at lysine 40.

### 2.2 Mechanics induce K40 acetylation of α‐tubulin in fibroblasts

**FIGURE 2:**
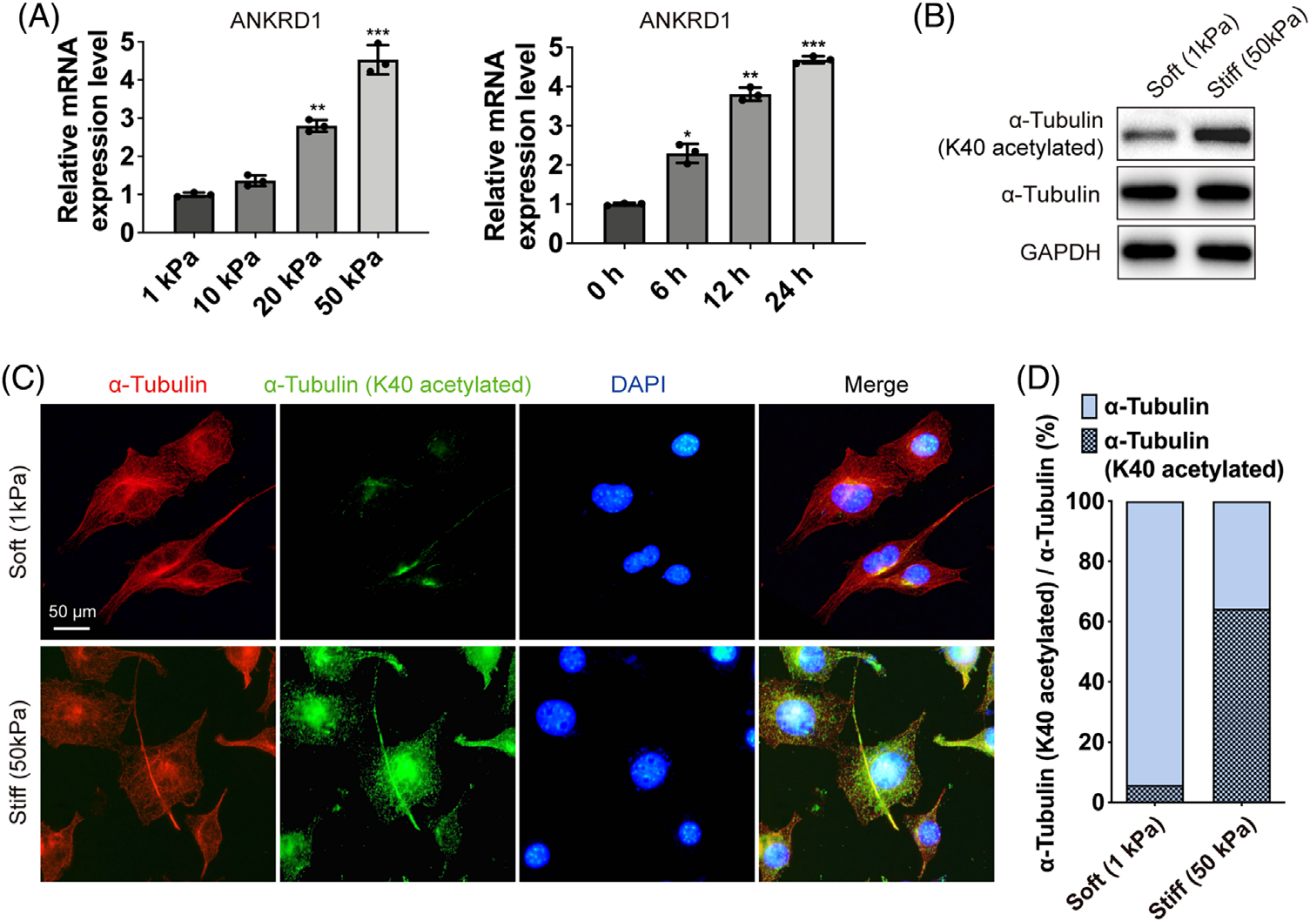
Expression of K40 acetylated α‐tubulin in polyacrylamide hydrogels of different substrate rigidities. (A) The relative mRNA expression of ANKRD1 from HDFs seeded on substrates with different rigidities and HDFs seeded on 50 kPa substrates with different timepoints was analyzed by qRT‐PCR. Statistical tests: ANOVA followed by Tukey test. (B) The protein levels of α‐tubulin (K40 acetylated) and total α‐tubulin in HDFs plated on soft (1 kPa) and stiff (50 kPa) hydrogels. (C) Images of immunofluorescent staining for α‐tubulin (K40 acetylated) (green) and total α‐tubulin (red) (scale bar = 50 μm). (D) The ratio of K40 acetylated α‐tubulin in HDFs plated on soft (1 kPa) and stiff (50 kPa) hydrogels. Data are presented as the means with SEs (*n* = 3 independent experiments).*p < 0.05, **p < 0.01, and ***p < 0.001.

To study the molecular mechanisms that regulate mechanics‐mediated skin fibrosis in vitro, the polyacrylamide (PA) hydrogels of different substrate rigidities were used to mimic the matrix stiffness of fibrotic skin.^22^, ^23^ Stiff substrates were produced to match Young's modulus of fibrotic skin tissues (50 kPa) and support cell fibrogenesis; a soft substrate modulus of 1 kPa was chosen to inhibit the activation of fibroblasts (Figure 2A).^24^, ^25^, ^26^ ANKRD1 was used as a mechanically sensitive gene to confirm that the mechanical loading environment has fully loaded on the fibroblasts (Figure 2A).^27^ Human dermal fibroblasts (HDFs) on stiff substrates exhibited higher levels of K40 acetylated α‐tubulin than cells on soft substrates (Figure 2B). Immunofluorescence analysis further confirmed this result, and the ratio of acetylated α‐tubulin at K40 increased from approximately 7%–63% with increased rigidity (Figure 2C,D). These results indicate that stiff hydrogels could induce α‐tubulin K40 acetylation levels in a mechanosensitive manner.

### 2.3 K40 acetylation of α‐tubulin is required for mechanics‐induced fibroblast activation

**FIGURE 3:**
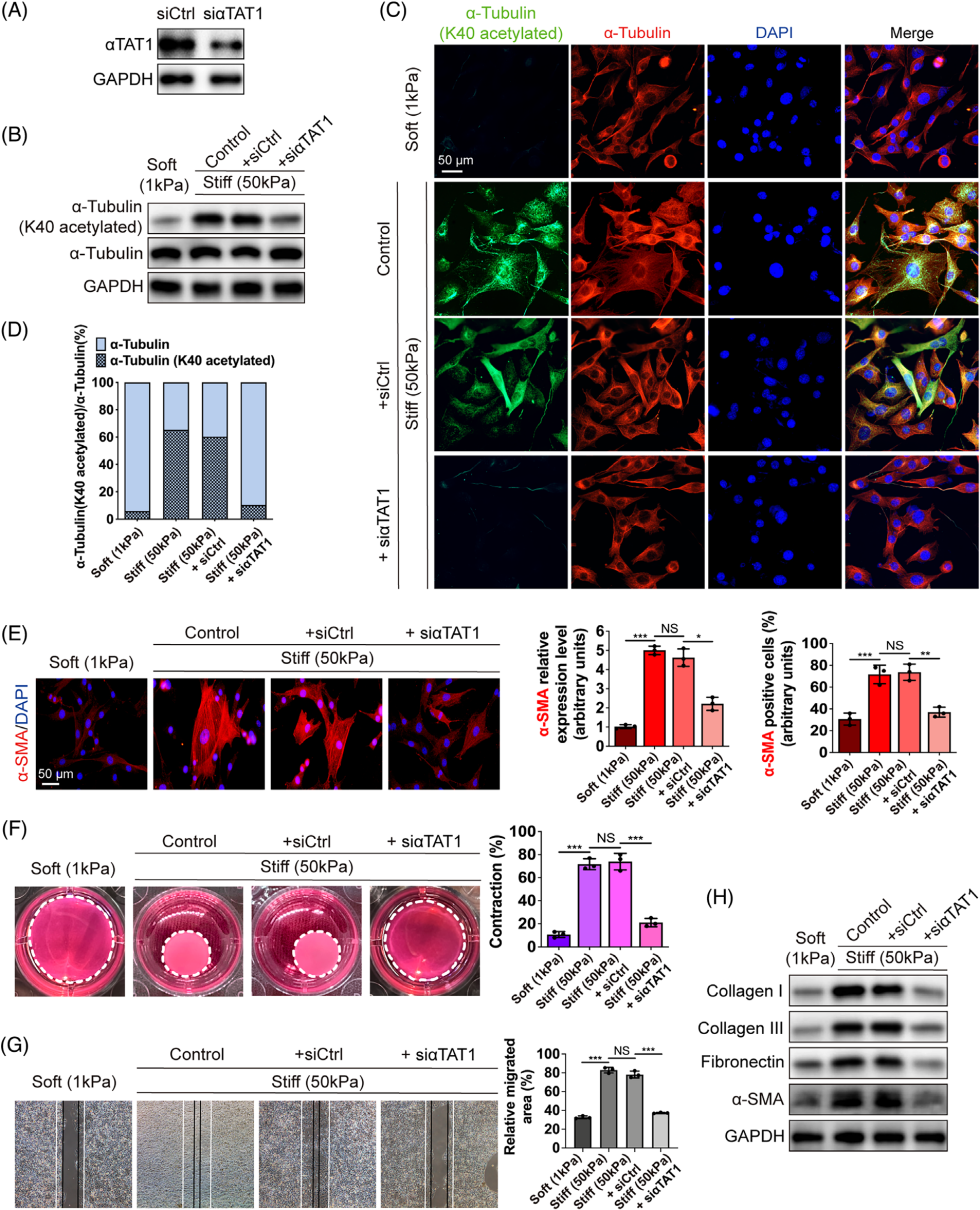
K40 acetylation of α‐tubulin is required for mechanics‐induced fibroblast activation. (A) Identification of siαTAT1 efficiency in HDFs cultured on stiff hydrogels (50 kPa). (B) Western blot assay of α‐tubulin (K40 acetylated) and total α‐tubulin in HDFs in different groups. (C, D) Images of immunofluorescent staining for α‐tubulin (K40 acetylated) (green) and total α‐tubulin (red) and the ratio of K40 acetylated α‐tubulin in different groups (scale bar = 50 μm). (E) Images of immunofluorescent staining for α‐SMA (red) in different groups and quantification of the expression of α‐SMA and the ratio of α‐SMA(+) cells in total fibroblasts (scale bar = 50 μm). Statistical tests: ANOVA followed by Tukey test. (F) Images and quantification of collagen lattice contraction assays in different groups. Dashed lines indicate the areas of the polymerized collagen gel at day 3. Statistical tests: ANOVA followed by Tukey test. (G) Images and quantification of wound healing assays in different groups. White lines indicate the edges of HDFs at 0 h, and the black lines indicate the edges of HDFs at 48 h. Statistical tests: ANOVA followed by Tukey test. (H) The protein levels of collagen type I, III, fibronectin, and α‐SMA in HDFs in different groups. Data are presented as the means with SEs (n = 3 independent experiments). NS = non significance, *p < 0.05, **p < 0.01, and ***p < 0.001.

The acetylation of microtubules is specifically mediated by αTAT1.^21^, ^28^ Therefore, we next investigated the impact of αTAT1 depletion (Figure 3A) on mechanics‐induced α‐tubulin K40 acetylation and fibroblast activation. Following αTAT1 knockdown (KD), the mechanics‐induced α‐tubulin K40 acetylation levels decreased correspondingly, that is, αTAT1‐depleted cells on stiff substrates showed an approximately 25% decrease in K40 acetylated α‐tubulin compared to that of siαTAT1 control cells (Figure 3B). Similarly, our immunofluorescence results presented a noticeable decrease in K40 acetylated α‐tubulin in αTAT1‐depleted cells plated on stiff substrates (Figure 3C), with the ratio of K40 acetylated α‐tubulin declining dramatically (Figure 3D).

Increased local tissue stiffness unlocks fibroblasts from their quiescent state and drives their differentiation into activated myofibroblasts.^29^ One of the key features of myofibroblasts is the neo‐expression of α‐SMA, an actin isoform typically present in vascular smooth muscle cells.^30^ Therefore, to determine the role of α‐tubulin acetylation in matrix stiffness‐induced fibroblast activation, the protein level of α‐SMA was investigated. In αTAT1‐KD fibroblasts plated on stiff substrates, the expression of α‐SMA and the ratio of α‐SMA positive cells in total fibroblasts were significantly downregulated compared to that of siαTAT1 control cells (Figure 3E). Cell migration and contraction have often been described as mechanosensitive cellular responses,^31^, ^32^ which are stimulated in fibrotic diseases with increased tissue stiffness.^33^ Next, we used a collagen lattice contraction assay and wound healing assay to study the contractile and migration ability of fibroblasts. The results revealed that fibroblasts plated on stiff substrates showed a greater ability to contract, which could be partly diminished by αTAT1 depletion (Figure 3F). In addition, we observed that the open area of the wound was markedly decreased in the stiff hydrogel group compared with the soft hydrogel group (Figure 3G). αTAT1 KD almost abolished the increased migration speed observed on stiff hydrogels (Figure 3G). Augmented synthesis and secretion of ECM proteins to facilitate tissue remodeling are shared outcomes of fibroblast activation,^6^ which forms a feed‐forward self‐reinforcing loop to enhance ECM stiffness.^4^ Our western blot assay showed that the crucial ECM proteins in skin fibrosis formation (collagen type I, III, and fibronectin) and the hallmark for myofibroblasts (α‐SMA), both evidently decreased with αTAT1 depletion (Figure 3H). These results imply that α‐tubulin K40 acetylation is required for matrix stiffness‐induced fibroblast activation.

### 2.4 Matrix stiffness‐induced α‐tubulin acetylation promotes fibroblast activation through YAP signaling

**FIGURE 4:**
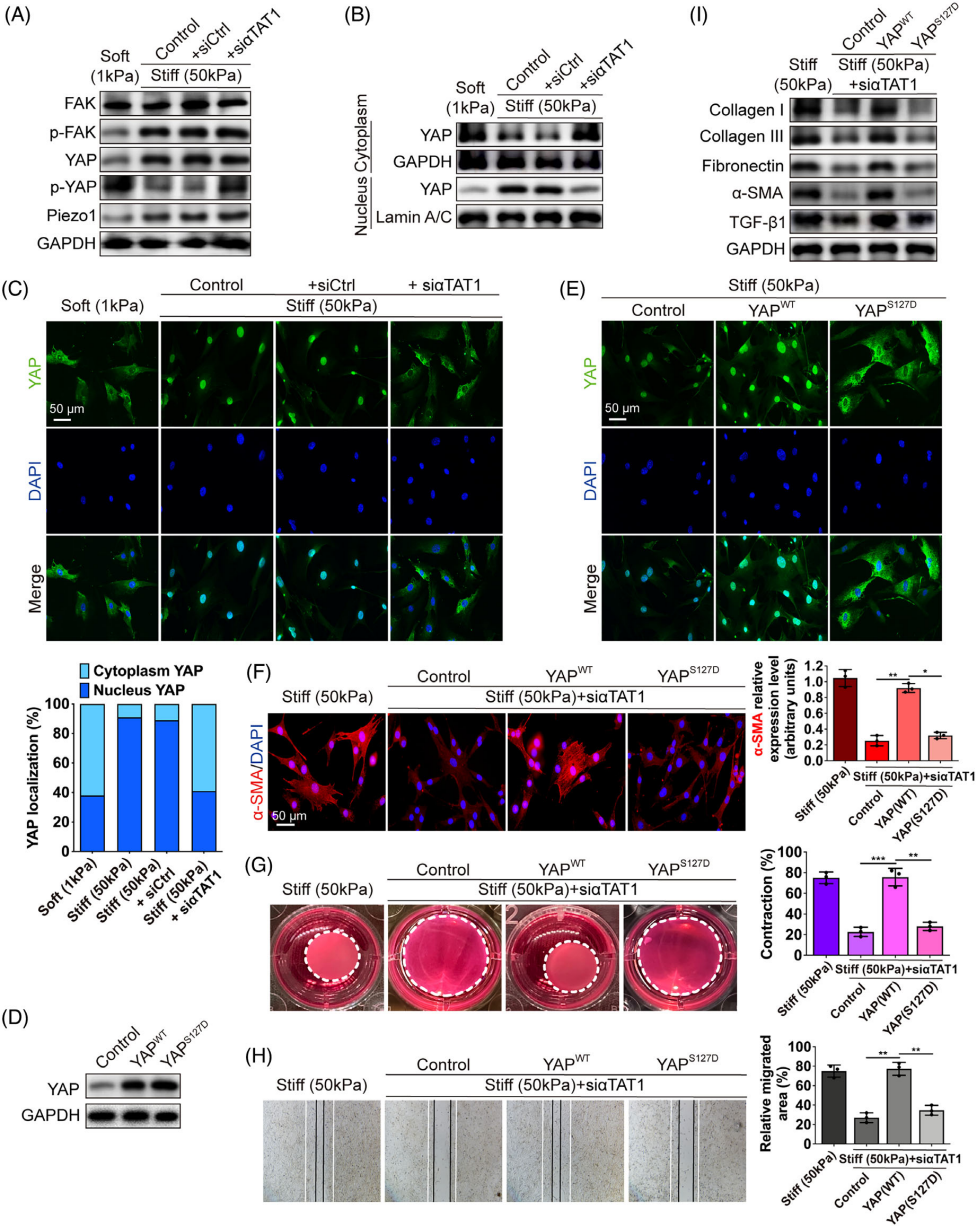
Matrix stiffness‐induced α‐tubulin acetylation promotes fibroblast activation through YAP signaling. (A) Western blot assay of Piezo1 and phosphorylated and total FAK and YAP in HDFs in different groups. (B) Western blot assay of YAP after abstraction of nuclear and cytoplasmic proteins. (C) Images of immunofluorescent staining for YAP (green) and the nuclear and cytoplasmic YAP ratio in different groups (scale bar = 50 μm). (D) Identification of YAP^WT^ and YAP^S127D^ overexpression in HDFs. (E) Images of immunofluorescent staining for YAP (green) in HDFs overexpressed with vector, YAP^WT^, and YAP^S127D^ (scale bar = 50 μm). (F) Images and quantification of immunofluorescent staining for α‐SMA (red) in different groups (scale bar = 50 μm). Statistical tests: ANOVA followed by Tukey test. (G) Images and quantification of collagen lattice contraction assay in different groups. Dashed lines indicate the areas of the polymerized collagen gel at day 3. Statistical tests: ANOVA followed by Tukey test. (H) Images and quantification of wound healing assays in different groups. White lines indicate the edges of HDFs at 0 h, and the black lines indicate the edges of HDFs at 48 h. Statistical tests: ANOVA followed by Tukey test. (I) The protein levels of collagen type I, III, fibronectin, α‐SMA, and TGF‐β1 in HDFs in different groups. Data are presented as the means with SEs (n = 3 independent experiments). NS = non significance, *p < 0.05, **p < 0.01, and ***p < 0.001.

The essential role of α‐tubulin acetylation in controlling cell contraction, migration, and protein deposition led us to further investigate the molecular mechanisms involved in this process. We examined the expression of the most‐studied elements in the mechanotransduction process, including focal adhesion kinase (FAK), YAP, and Piezo1, whose protein expression levels showed a significant upregulation on stiff substrates (Figure 4A). However, the expression of these proteins did not respond to αTAT1 depletion (Figure 4A). Interestingly, phosphorylated YAP declined on stiff substrates and resumed to the level of soft substrates upon αTAT1 depletion (Figure 4A). We next investigated the subcellular distribution of YAP. YAP translocated into the nucleus when the substrate rigidity increased, as expected, and αTAT1 depletion abolished YAP nuclear retention (Figure 4B). Immunofluorescence analysis gave us a more direct view of YAP localization: on stiff substrates, cytoplasmic YAP was clearly reduced, and the nuclear accumulation of YAP was reversed by αTAT1 depletion (Figure 4C). These results indicate that α‐tubulin K40 acetylation is required for mechanics‐induced YAP activation.

To further prove that acetylated K40 α‐tubulin promotes matrix stiffness‐induced fibroblast activation through YAP dephosphorylation and nuclear accumulation, we transduced HDFs with a plasmid expressing wild‐type YAP or YAP S127D^34^ (Figure 4D). Substitution of serine 127 with aspartic acid (S127D) generates a YAP protein that mimics phosphorylated YAP, loses its ability to bind with 14‐3‐3 protein,^35^ and is restricted to the cytoplasm. Immunofluorescent staining confirmed that HDFs on stiff substrates with overexpressed wildtype YAP were mainly located in the nucleus whereas overexpressed S127D YAP was restricted to the cytoplasm (Figure 4E). Immunofluorescence analysis showed the overexpressed wild‐type YAP successfully activated αTAT1‐depleted fibroblasts with elevated expression of α‐SMA, however, the S127D mutant failed to trigger the expression of α‐SMA (Figure 4F). Contraction assays and wound healing assays confirmed that fibroblasts treated with wild‐type YAP contracted more significantly and migrated faster, and those treated with the S127D mutant showed no obvious difference (Figure 4G,H). Our data also demonstrated that fibroblasts treated with wild‐type YAP synthesized and deposited more collagen type I, III, and fibronectin than those treated with the S127D mutant (Figure 4I). Additionally, the expression level of α‐SMA and transforming growth factor‐β1 (TGF‐β1) was elevated with overexpressed wildtype YAP, but not S127D mutant YAP (Figure 4I).

Collectively, these observations imply the critical role of nuclear YAP and indicate that acetylated α‐tubulin at K40 promotes fibroblast activation by directly dephosphorylating YAP and maintaining its nuclear retention. Depletion of αTAT1 frees YAP from nuclear accumulation, uncouples cells from YAP‐dependent mechanotransduction, and eventually suppresses the matrix stiffness‐induced fibroblast activation.

### 2.5 Reducing acetylated α‐tubulin at K40 attenuates bleomycin‐induced skin fibrosis

**FIGURE 5:**
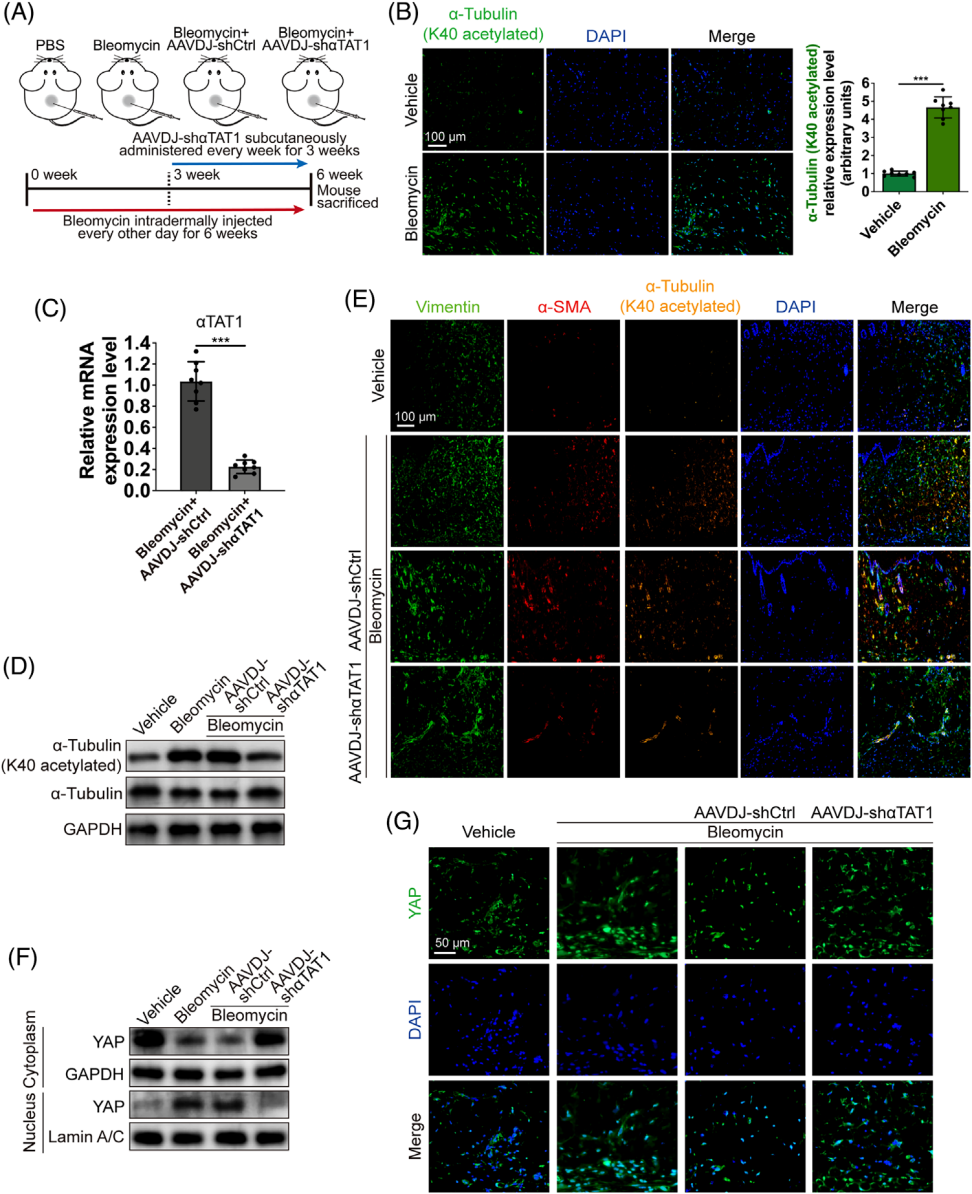
Reducing acetylated α‐tubulin at K40 hinders YAP cytoplasm‐nucleus shuttle in bleomycin‐induced skin fibrosis model. (A) Diagram of bleomycin‐induced skin fibrosis animal model and therapeutic intervention. (B) Images and quantification of immunofluorescent staining for α‐tubulin (K40 acetylated) (green) in bleomycin‐induced mouse skin fibrosis tissue and normal skin (scale bar = 100 μm). Statistical tests: *t*‐test. (C) Identification of AAVDJ‐shαTAT1 efficiency in mouse skin tissues. Statistical tests: *t*‐test. (D) Western blot assay of α‐tubulin (K40 acetylated) and total α‐tubulin in different groups. (E) Images of immunofluorescent staining for α‐tubulin (K40 acetylated) (yellow), α‐SMA (red), and Vimentin (green) in different groups (scale bar = 100 μm). (F) Western blot assay of YAP after abstraction of nuclear and cytoplasmic proteins. (G) Images of immunofluorescent staining for YAP (green) in different groups (scale bar = 50 μm). Data are presented as the mean ± SD (n = 8 biologically independent animals). ***p < 0.001.

To evaluate the crucial role of α‐tubulin K40 acetylation in skin fibrosis, we used a bleomycin‐induced murine model of dermal fibrosis^36^ (Figure 5A). Following the trend of human fibrotic skin tissues, the K40 acetylation level of α‐tubulin was markedly higher in bleomycin‐induced mouse skin fibrosis tissue than in normal skin (Figure 5B). We next subcutaneously (that is, locally) administered AAVDJ to construct short hairpin RNA (shRNA) of αTAT1 into the related skin area^37^ (Figure 5A). quantitative real‐time polymerase chain reaction (qRT‐PCR) confirmed the KD of αTAT1, and the reduction in acetylated α‐tubulin at K40 was verified by western blotting (Figure 5C, D).

**FIGURE 6:**
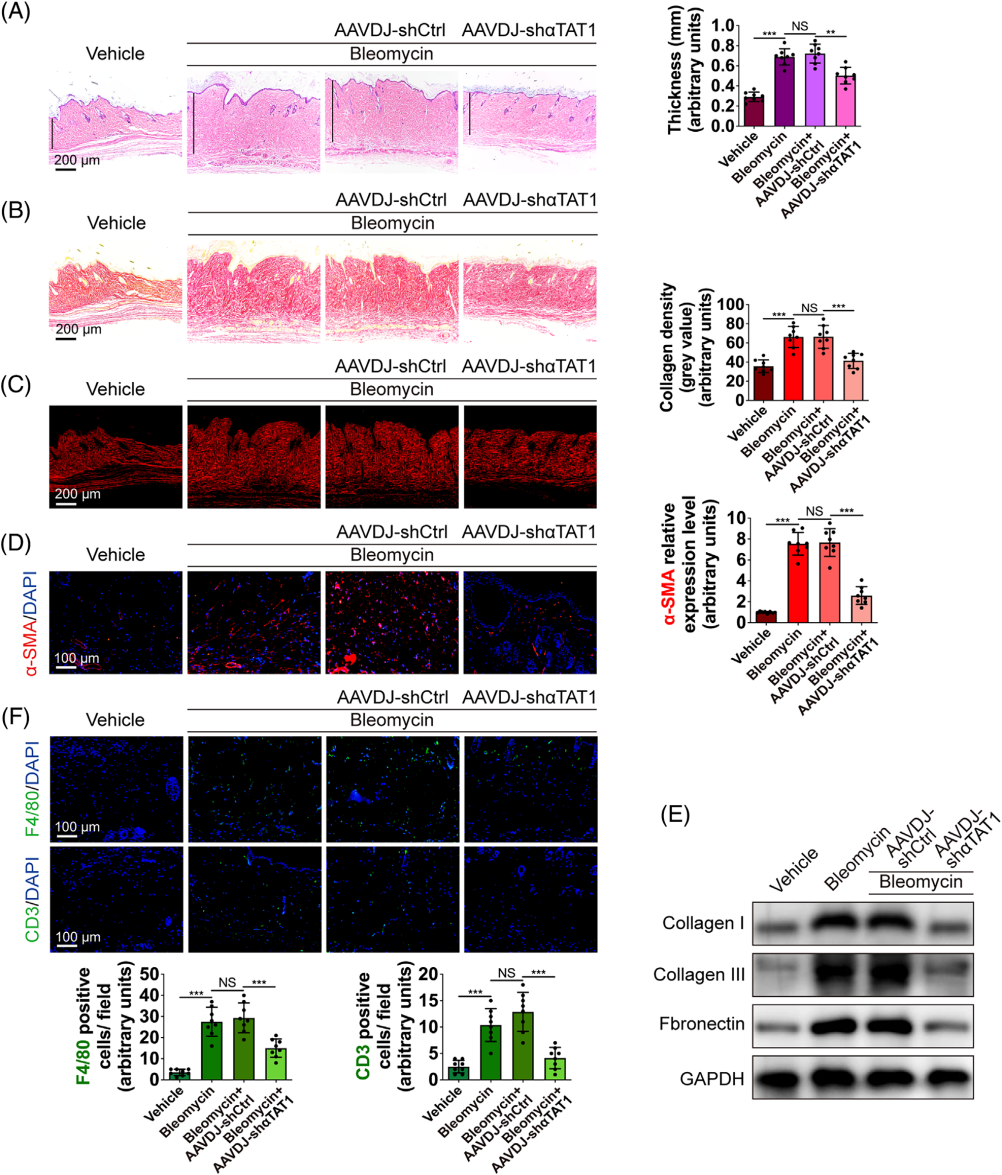
Diminishing acetylated α‐tubulin at K40 prevents bleomycin‐induced skin fibrosis. (A) Images of H&E staining and quantification in different groups (scale bar = 200 μm). Statistical tests: ANOVA followed by Tukey test. (B, C) Images of picrosirius red staining under ordinary and polarized light and collagen density quantification in different groups (scale bar = 200 μm). Statistical tests: ANOVA followed by Tukey test. (D) Images and quantification of immunofluorescent staining for α‐SMA (red) in different groups (scale bar = 100 μm). Statistical tests: ANOVA followed by Tukey test. (E) The protein levels of collagen type I, III, and fibronectin in different groups. (F) Images and quantification of immunofluorescent staining for F4/80 (green) and CD3 (green) in different groups (scale bar = 100 μm). Statistical tests: ANOVA followed by Tukey test. Data are presented as the mean ± SD (n = 8 biologically independent animals). NS = non significance, **p < 0.01, and ***p < 0.001.

In fibrotic skin tissues, the acetylation of α‐tubulin at K40 mainly occurred in α‐SMA(+) myofibroblasts (Figure 5E), as we observed in human HS tissues. And the intervention of AAVDJ‐shαTAT1 could significantly reduce the expression of K40 acetylated α‐tubulin as well as α‐SMA (Figure 5E). Western blot assay following extraction of nuclear and cytoplasm proteins showed that YAP displayed a translocation into the nucleus in murine fibrotic skin tissues, and the cytoplasm‐nucleus shuttle was blocked by AAV‐shαTAT1 intervention (Figure 5F), the immunofluorescence yielded the same conclusion as a Western blot (Figure 5G). Histological analysis demonstrated that AAVDJ‐shαTAT1 treated mice showed attenuated fibrosis formation with significantly reduced dermal thickness compared with the AAVDJ‐shCtrl group (Figure 6A). To better evaluate the degree of skin fibrosis formation, we further analyzed the collagen density of skin sections following the staining of picrosirius red. As shown in Figure 6B,C, bleomycin‐treated mice demonstrated markedly increased collagen density in the skin tissues, and skin from αTAT1‐KD mice showed significantly rescued collagen deposition. Our in vivo study also demonstrated an effect that consistent with the in vitro studies, in that αTAT1‐KD significantly downregulated the α‐SMA(+) myofibroblast population and excessive deposition of ECM proteins (Figure 6D,E).

Given that immune infiltrates are another significant element in the progression of bleomycin‐induced skin fibrosis. We examined the macrophages with F4/80 and T cells with CD3 through immunofluorescence. Our results revealed an increase in the number of F4/80(+) macrophages, as well as the number of CD3(+) T cells in the bleomycin‐treated murine skin (Figure 6F). Moreover, the intervention of AAVDJ‐shαTAT1 downregulated the number of immunoreactive cells for CD3 and F4/80 (Figure 6F), suggesting the depletion of αTAT1 and deactivation of YAP could influence the immune microenvironment.

## 3 DISCUSSION AND CONCLUSION

The matrix stiffness microenvironment regulates skin fibrosis development, but the underlying mechanisms remain to be fully clarified. Our results show that microtubules sense the change in substrate rigidity and in turn play a central role in mechanotransduction by participating in mechanosensitive cellular responses, including translocation of the transcriptional coactivator YAP, the activation of fibroblasts, generation of traction forces, and cell migration. Our results point to a mechanism by which posttranslational modification of α‐tubulin, K40 acetylation in particular, is stimulated under the stiffened mechanical loading of skin fibrosis and controls the dephosphorylation of YAP S127 to induce its nuclear retention and function. Furthermore, we demonstrated that the depletion of αTAT1 could ameliorate skin fibrosis formation.

Microtubules are dynamic polymers that self‐organize and constitute the architecture of the cytoskeleton. The “tubulin code”, which is defined by tubulin isoforms and PTMs, modulates the structure of microtubules to support the diverse morphology of microtubule arrays across different cell types and developmental stages.^38^ Tubulin is further diversified with many evolutionarily conserved but chemically distinct PTMs, including phosphorylation, detyrosination, acetylation, methylation, palmitoylation, polyamination, and so forth, many of which vary widely between cell types.^39^ Our results prove that K40 acetylation of α‐tubulin is upregulated in fibrotic skin tissues, as well as fibroblasts, on PA hydrogels with high substrate rigidity. Previous studies showed that astrocytes and human umbilical vein endothelial cells on stiff substrates exhibited higher levels of acetylated α‐tubulin.^23^ Laura et al. seeded enzymatically acetylated and deacetylated microtubules onto micropatterns, subjected these microtubules to transient hydrodynamic flow, and reconstituted microtubule breakage.^18^ They found that in this in vitro model of microtubule self‐repair, acetylated tubulins acquired resistance and protected microtubules from mechanical breakage.^40^ These investigations suggest that the acetylation of tubulins stabilizes microtubules, which is possibly a universally existing phenomenon in response to mechanical stimulation, and consequently has the potential to be an intervention target for mechanotransduction.

When we talk about tubulin acetylation, we refer to the acetylation of Lys 40 in α‐tubulin in most cases. Although acetylation of other sites has been identified by mass spectrometry,^41^ K40 of α‐tubulin has received particular attention. αTAT1 is responsible for almost all acetylation on K40 of α‐tubulin in every organism studied since its discovery.^42^ We have shown that the depletion of αTAT1 remarkably regulated the phosphorylation level of YAP and controlled its cytoplasm‐nuclear shuttle in fibroblasts. And another study demonstrated similar results in astrocytes with αTAT1‐2 depletion.^23^ How acetylated α‐tubulins participate in the process of YAP phosphorylation and nuclear transport needs further investigation. Microtubules construct a motor‐driven intracellular transport network and interact with various accessory proteins named microtubule‐associated proteins (MAPs).^43^ MAP guanine nucleotide exchange factor‐H1 (GEF‐H1), a microtubule‐bound RhoGEF that is released from microtubules upon acetylation to trigger the Rho/ROCK signaling cascade,^44^, ^45^ could be one of the possible intermediate agents between tubulin acetylation and mechanosensitive YAP activation.^46^ The widely accepted model for YAP shuttling and functioning is that the phosphorylation at S127 of YAP provides a binding site for 14‐3‐3 protein to cause its spatial separation from TEAD.^47^ However, a recent study found that YAP could be the cargo of acetylated microtubules and be directly transported into the nucleus in a dynein‐mediated manner,^48^ indicating the existence of alternative pathways to regulate YAP activity. Regardless, acetylated α‐tubulins remain the activator for the mechanosensitive YAP cytoplasm‐nuclear shuttle and its transcriptional activation. The reduction in the α‐tubulin acetylation level mediated by αTAT1 depletion discharges YAP from mechanotransduction and blocks the cell from sensing mechanical tension.

Following the acetylation of α‐tubulin, YAP was translocated into the nucleus and was put into function to activate downstream signaling pathways. Our results displayed a connection between the nuclear YAP and the upregulation of TGF‐β1, which were in correspondence with previously published studies that knocked out of LATS1/2 could lead to decreased expression of phosphorylated YAP, which could cause increased expression of TGF‐β in mouse neuroepithelium and liver cells.^49^, ^50^ Other studies also showed that when the activity of YAP was inhibited with Verteporfin, the TGF‐β pathway was hindered in fibroblast,^51^ and the secretion of TGF‐β by macrophages was diminished.^52^ These results suggest a possible crosstalk between Hippo and TGF‐β pathways where dephosphorylated YAP could translocate into the nucleus to activate the TGF‐β signaling pathway.

Compounds that disrupt microtubule dynamics are one of the most successful and widely used chemotherapeutic agents against cancer.^53^, ^54^ The extensive experience we gained from tumor intervention might help us to develop antifibrosis therapies targeting microtubules. αTAT1 is a highly conserved enzyme that specifically transfers aminoacyl groups. The αTAT1 knockout mice are viable and display no overt phenotypes,^55^ suggesting that αTAT1 did not function under physiological conditions. These characteristics make αTAT1 suitable as an intervention target. Our results prove that the depletion of αTAT1 was effective in ameliorating fibrosis formation and development. A recent study constructed pharmacophore anchor models of αTAT1 for drug design and proposed ten potential inhibitors of αTAT1.^56^ Although this study is merely an early‐stage, theoretical notion that lacks in vitro and in vivo verification, it shows the possibility of pharmacological inhibition of αTAT1 activity, reduction of α‐tubulin acetylation levels, and even disruption of the mechanotransduction process in diseases including but not limited to skin fibrosis formation.

Our results presented that the acetylation of α‐tubulin could activate the mechanosensitive Hippo‐YAP signaling pathway. It is also crucial to clarify how the intermediate protein attaches to or detaches from the acetylated tubulin cytoskeleton, and thus has the chance to regulate downstream signaling pathways including YAP. Our work merely revealed the tip of the iceberg, and further studies are needed to uncover the molecular mechanism of the mechanosensitive regulatory network centered on tubulin PTMs. We emphasized the significant role of acetylated α‐tubulin in fibroblasts. However, fibrosis is a highly dynamic process that involves not only fibroblasts’ activation but also epithelial‐mesenchymal transition and immune responses.^57^, ^58^ Our results showed that in bleomycin‐induced skin fibrosis, the depletion of αTAT1 and associated YAP cytoplasmic retention has an influence on the immunological infiltrate process, indicating the importance of macrophages and T cells in the fibroinflammatory program. Previous studies also demonstrated that the Hippo signaling pathway could not only affect the nature of fibroblasts but also the polarization as well as infiltration of macrophages.^59^, ^60^ Substantial progress in the understanding of the complex network of fibrogenesis needs to be made, which could minimize the translational gap for effective treatments.

In conclusion, our results highlight the crucial role of matrix stiffness‐induced α‐tubulin K40 acetylation in skin fibrosis formation. We revealed that α‐tubulin acetylation is required for fibroblast activation by regulating the mechanosensitive YAP S127 dephosphorylation, with phenotypes that include enhanced contraction, migration, and ECM deposition. Our findings also imply that the depletion of αTAT1 shows positive therapeutic effects on skin fibrosis formation. Our study emphasizes the significance of α‐tubulin acetylation in diseases involving aberrant mechanical tension, which will hopefully aid in the transfer of knowledge from the lab bench to the bedside.

## 4 MATERIALS AND METHODS

### 4.1 Patient samples

The human hypertrophic scar tissues and corresponding normal skin tissues were obtained from 10 people (Table S1). All the samples were obtained from Shanghai Ninth People's Hospital with ethics approval from the Human Research Ethics Committee of Shanghai Ninth People's Hospital, Shanghai Jiao Tong University School of Medicine in accordance with the principles of the Declaration of Helsinki (approval number SH94‐2019‐TK101‐1). Written informed consent was obtained from patients.

### 4.2 Animals

C57BL/6 mice were obtained from Shanghai Laboratory Animal Center (Slac, Shanghai, China) at an age of 8 weeks. The use of animals was approved by the Committee on the Ethics of Animal Experiments of Shanghai Ninth People's Hospital, Shanghai Jiao Tong University School of Medicine (approval number SH94H‐2019‐A302‐1).

Mice were randomly divided into four groups, and each group contained 8 mice: (1) phosphate‐buffered saline (PBS)‐treated control mice; (2) skin fibrosis mice; (3) skin fibrosis mice intervened with AAVDJ‐shCtrl; (4) skin fibrosis mice intervened with AAVDJ‐shαTAT1. Bleomycin was used to induce skin fibrosis according to previously published protocols.^36^, ^61^ In brief, bleomycin (Cat.#67763‐87‐5, MedChemExpress, China) was intradermally injected into the upper back of the mouse after fur removal. 100 μl bleomycin solution (1 unit USP/ml) was delivered every other day. Three weeks after the first injection, mice should present an obvious change of skin fibrosis and be suitable for further therapeutic intervention.

### 4.3 Atomic force microscopy

The measurements of Young's modulus of fresh tissues were carried out using Dimension FastScan Bio (Bruker, MA, USA) by the Instrumental Analysis Center of Shanghai Jiao Tong University following a standardized procedure. Data were analyzed by NanoScope Analysis v.180r1 (Veeco Instruments Inc., NY, USA) according to the manufacturer's instructions.

### 4.4 Preparation of collagen‐conjugated PA hydrogels

The protocol used to prepare hydrogels was adapted from references.^23^, ^31^ The 0.75 mm parallel glass plates were washed with absolute ethanol and dried. A 5 ml solution of PA was prepared. The proportions of acrylamide and bisacrylamide in the solution define the rigidity of the hydrogel. Then, 50 μl 10% ammonium persulfate (APS) and 5 μl tetramethylethylenediamine were added and the solution was mixed well. The solution was added to each set of the gel casting plates and was allowed to polymerize for 1 h at room temperature. The parallel glass plates were immersed into *N*‐2‐hydroxyethylpiperazine‐*N*'‐2‐ethanesulfonic acid (HEPES) to gently detach the top glass. The polymerized hydrogel was cut, transferred into 6‐well plates, and washed with 10 mM HEPES twice. The hydrogel was then activated under ultraviolet light for 10 min using 5 mg/ml sulpho‐SANPAH (Cat.#ab145610, Abcam, Cambridge, UK) and washed with 50 mM HEPES twice. The hydrogels were then coated with 100 μg/ml of rat tail collagen I (Cat.#354236, Corning, NY, USA) overnight at 4°C. The excess collagen was washed out with PBS before plating cells.

### 4.5 Primary HDFs

HDFs were harvested from the dermis of discarded skin samples derived from circumcisions of healthy males, with institutional approvals acquired and written informed consent obtained from patients. Adipose and deeper dermal tissues were removed as much as possible. Skin tissues were then cut into approximately 10 mm^2^ pieces and digested at 37°C in 0.3% Dispase II (Cat.#D4693; Sigma‐Aldrich, MA, USA) overnight. The epidermis was gently detached. And the rest skin tissue was digested at 37°C in 0.3% Collagenase II (Cat.#C2‐28; Sigma‐Aldrich) for 6 h. After filtering through sterilized gauze, cells were centrifugated, collected, and plated into 10 cm dishes. A DMEM (Cat.#11995065; Gibco, NY, USA) containing 10% fetal bovine serum (FBS) (Cat.#10437028; Gibco) and 1% penicillin‐streptomycin (Cat.#B1351101; BIOEXPLORER, CO, USA) were used for cell culture. The medium was changed every 3 days. The HDFs were cultured on substrates with different rigidities and were collected for further analysis after 24‐h culture.

### 4.6 Cell nucleofection

For αTAT1 silencing, cells were transfected in 6‐well plates with 25 nM siRNA (ON‐TARGETplus SMARTpool; Dharmacon, CO, USA) using Lipofectamine RNAiMAX transfection reagent (Cat.#13778; Invitrogen, CA, USA) according to the manufacturer's instructions. Experiments were carried out 2 days post‐transfection and comparable protein silencing was observed. The sequences were as follows:

Human αTAT1 si‐1: 5′‐GUAGCUAGGUCCCGAUAUA‐3′,
Human αΤAT1 si‐2: 5′‐GAGUAUAGCUAGAUCCCUU‐3′,
Human αTAT1 si‐3: 5′‐GGGAAACUCACCAGAACGA‐3′,
Human αTAT1 si‐4: 5′‐CUUGUGAGAUUGUCGAGAU‐3′.

### 4.7 Plasmid preparation and transfection

The lentiviral vectors overexpressing YAP^WT^ and YAP^S127D^ were purchased from Genechem (Shanghai, China). The vectors used were GV358. The mutant YAP harboring the S127D (serine to aspartic acid at residue 127) was inserted into the GV358 vector and constructed the recombinant lentiviral plasmid. The primer sequences for YAP1 were as follows:

Human YAP1 forward: 5′‐GAGGATCCCCGGGTACCGGTCGCCACCATGGATCCCGGGCAGCAGCCGC‐3′,
Human YAP1 reverse: 5′‐TCCTTGTAGTCCATACCTAACCATGTAAGAAAGCTTTC‐3′.

The clones that stably expressed YAP^WT^, YAP^S127D^, and empty vector control were selected using 2 μg/ml puromycin.

### 4.8 Total RNA extraction and qRT‐PCR

RNA was extracted from the culture samples using TRIzol Reagent (Cat.#15596018, Invitrogen, CA, USA) following the manufacturer's instructions. The quantity and purity of extracted RNA were measured by spectrophotometry (NanoDrop2000, Thermo Fisher, MA, USA). Complementary DNA was synthesized from total RNA using the RevertAid Reverse Transcriptase (Cat.#EP0441, Thermo Fisher, MA, USA). Relative mRNA expression levels were measured using qRT‐PCR, normalized to Gapdh, using TB Green Premix Ex Taq II (Cat.#RR820A, Takara, Shiga, Japan) and LightCycler 480 System (Roche, IN, USA). The primers used in our study were as follows:

Human ANKRD1 forward: 5′‐GCCTACGTTTCTGAAGGCTG‐3′,
Human ANKRD1 reverse: 5′‐GTGGATTCAAGCATATCACGGAA‐3′,
Human GAPDH forward: 5′‐GGAGCGAGATCCCTCCAAAAT‐3′,
Human GAPDH reverse: 5′‐GGCTGTTGTCATACTTCTCATGG‐3′,
Mouse αTAT1 forward: 5′‐AGCAACCGGCACGTTATTTAC‐3′,
Mouse αTAT1 reverse: 5′‐GCAAAGGGGTTCTACCTCATTGT‐3′,
Mouse Gapdh forward: 5′‐AGGTCGGTGTGAACGGATTTG‐3′,
Mouse Gapdh reverse: 5′‐TGTAGACCATGTAGTTGAGGTCA‐3′.

### 4.9 Western blot

Cell lysates were obtained with ice‐cold RIPA lysis buffer, with the addition of 1 mM PMSF. The extraction of nuclear and cytoplasmic proteins was performed according to the manufacturer's instructions (Cat.#AR0106, Boster, Wuhan, China). Sample concentration was detected by bicinchoninic acid assay, and samples were boiled for 15 min at 98°C. 20 ng protein lysate was run on a 10% SDS‐PAGE gel at 80 V for 45 min and then 120 V for 60 min. The transfer took place at 300 mA for 120 min on PVDF membranes. Membranes were blocked at room temperature with 5% milk for 60 min. The membranes were then incubated with primary antibodies overnight at 4°C and with horseradish peroxidase (HRP)‐conjugated secondary antibodies at room temperature for 1 h. Bands were revealed using ECL chemoluminescent substrate (Cat.#E41201; Vazyme, Nanjing, China) and Tanon 4600 Imaging System (Tanon, Shanghai, China). Quantitative analysis was performed by ImageJ.

The primary antibodies used were: anti‐alpha Tubulin (acetyl K40) (Cat.#ab179484, Abcam, 1:2000), anti‐alpha Tubulin (Cat.#ab7291, Abcam, 1:5000), anti‐GAPDH (Cat.#ab8245, Abcam, 1:5000), anti‐alpha TAT (Cat.#28828, ProteinTech, 1:1000), anti‐Collagen I (Cat.#ab260043, Abcam, 1:1000), anti‐Collagen III (Cat.#22734, ProteinTech, 1:500), anti‐alpha SMA (Cat.#ab124964, Abcam, 1:10000), anti‐Fibronectin (Cat.#ab2413, Abcam, 1:2000), anti‐FAK (Cat.# ab40794, Abcam, 1:1000), anti‐FAK (phospho Y397) (Cat.# ab 81298 Abcam, 1:1000), anti‐YAP1 (Cat.#A11264, ABclonal, 1:1000), anti‐Phospho‐YAP (Ser127) (Cat.#13008, CST, 1:1000), anti‐Piezo1 (Cat.#NBP1‐78537, Novus, 1:500), anti‐Lamin A + Lamin C (Cat.#ab108595, Abcam, 1:5000), anti‐TGF‐β1 (Cat.#21898‐1‐AP, ProteinTech, 1:2000). The secondary antibodies used were: goat anti‐rabbit IgG HRP (Cat.#ab6721, Abcam, 1:10000), anti‐mouse IgG, HRP‐linked (Cat.#7076, CST, 1:2000).

### 4.10 Wound healing assay

Wound healing assays were performed using a 2‐well culture insert (Cat.#80209; ibidi, Germany) following the manufacturer's instructions. Cells were cultured in DMEM without FBS. The migrated cells were imaged using a Nikon Eclipse E800 microscope (Nikon, Tokyo, Japan) every 6 h. The relative migrated area was analyzed using ImageJ.

### 4.11 Collagen lattice contraction assay

Cells were resuspended in Collagen I (collagen concentration at 1 mg/ml, pH = 7; Cat.#Biocoat 354236; Corning, NY, USA) and gently mixed. A 24‐well plate was plated with 800 μl of the suspension in each well. The collagen gel was polymerized after 30 min, 37°C incubation. After polymerization, 1 ml DMEM supplemented with 10% FBS was added to each well and the gels were eased from the wells. The gels were photographed after 3 days. The contraction area and percentage were analyzed using ImageJ.

### 4.12 Immunostaining

4% paraformaldehyde was used for cell sample and tissue sample fixation. The tissue samples were paraffin‐embedded, frozen, and sliced. The samples were permeabilized for 5 min with 0.1% Triton X‐100. The coverslips were blocked for 1 h in PBS supplemented with 5% bovine serum albumin at room temperature. The same solution was used for primary and secondary antibody incubation. The nucleus was stained with DAPI and the coverslips were mounted with antifade medium. Images were obtained using a Nikon Ni‐U microscope and DS‐Ri2 camera (Nikon). Quantitative analysis was performed by ImageJ.

The primary antibodies used were: anti‐alpha Tubulin (acetyl K40) (Cat.#ab179484, Abcam, 1:400), anti‐alpha Tubulin (Cat.#ab7291, Abcam, 1:200), anti‐alpha SMA (Cat.#67735, ProteinTech, 1:200), anti‐Vimentin (Cat.#ab92547, Abcam, 1:500), anti‐YAP1 (Cat.#A11264, ABclonal, 1:100), anti‐F4/80 (Cat.#27044‐1‐AP, ProteinTech, 1:200), anti‐CD3 (Cat.#ab16669, Abcam, 1:200). The secondary antibodies used were: donkey anti‐mouse IgG (H+L) secondary antibody, Alexa Fluor Plus 555 (Cat.#A32773, Invitrogen, 1:500), donkey anti‐rabbit IgG (H+L) secondary antibody, Alexa Fluor 488 (Cat.#A21206, Invitrogen, 1:500).

### 4.13 AAV transduction

The shαTAT1 and negative control AAVs were purchased from Ji Manchu Biotech (Shanghai) Co., Ltd. The vector used was PGMAAV‐10261. The target sequences were as follows:

Negative control: TTCTCCGAACGTGTCACGT;
αTAT1 shRNA: GCAACCGGCACGTTATTTACA.

Note that, 100 μl of AAVDJ‐shαTAT1 or AAVDJ‐shCtrl was subcutaneously injected into the fibrotic mice dorsal skin. Three weeks after the AAV administration, mice were euthanized by intraperitoneal injection of avertin and sacrificed. Dorsal skin samples were collected.

### 4.14 Histological staining

Paraformaldehyde‐fixed mouse skin samples were paraffin‐embedded and sliced into five μm sections. Slides were deparaffinized in xylene and rehydrated through a series of graded ethanol until water. The serial sections were stained with hematoxylin and eosin (H&E) and picosirius red (Solarbio, Beijing, China). The sections were imaged with a Nikon Ni‐U microscope and DS‐Ri2 camera (Nikon). Quantitative analysis was performed by ImageJ.

### 4.15 Polychromatic immunofluorescence staining

The prepared skin sections were stained using the four‐color multi‐fluorescent immunohistochemical staining kit (Cat.#Abs50028, Absin, Shanghai, China) based on the Tyramide signal amplification (TSA) technology following the manufacturer's instructions. The antibodies involved include anti‐alpha Tubulin (acetyl K40) (Cat.#ab179484, Abcam, 1:500), anti‐alpha SMA (Cat.#ab124964, Abcam, 1:500), anti‐Vimentin (Cat.#ab92547, Abcam, 1:500). The nucleus was stained with DAPI and the coverslips were mounted with antifade medium. Images were captured using a Nikon Ni‐U microscope and DS‐Ri2 camera (Nikon).

### 4.16 Statistical analysis

IBM SPSS Statistics was used for the analysis of statistical data. *P* < 0.05 was considered to discriminate significance. Shapiro‐Wilk test was performed for testing whether the data followed a normal distribution. An independent‐samples *t*‐test was used for comparisons between the two groups. Levene's test was performed for testing the equality of variances. One‐way analysis of variance (ANOVA) followed by a post hoc test (Tukey test) was used to compare multiple groups. At least three independent replicates were used for each experiment.

## AUTHOR CONTRIBUTIONS

Conceptualization: Y.Z., Q.L., and D.W.; Data Curation: D.W., Y.G., Y.L., and Y.Z.; Formal Analysis: D.W., Y.G., Y.L., Y.Z., and Q.L.; Investigation: D.W., Y.G., Y.L., C.H., J.S., L.H., and Y.L.; Funding acquisition: Y.G., Y.Z., and Q.L.; Supervision: Y.Z. and Q.L.; Writing‐Original Draft Preparation: D.W. and Y.Z.; Writing‐Review and Editing: D.W., Y.G., Y.Z., and Q.L. All authors read and approved the final paper.

## CONFLICT OF INTEREST STATEMENT

The authors declare no conflict of interest.

## ETHICS STATEMENT

Human tissues: All performances were approved by the Human Research Ethics Committee of Shanghai Ninth People's Hospital, Shanghai Jiao Tong University School of Medicine (approval number SH94‐2019‐TK101‐1). Written informed consent was obtained from patients.

Animals: All mice experimental procedures were approved by the Committee on the Ethics of Animal Experiments of Shanghai Ninth People's Hospital, Shanghai Jiao Tong University School of Medicine (approval number SH94H‐2019‐A302‐1).

## Supporting information

Supporting Information

## Data Availability

The data that support the findings of this study are available from the corresponding author upon reasonable request.
