# Supplementary material for: Matrix stiffness‐induced α‐tubulin acetylation is required for skin fibrosis formation through activation of Yes‐associated protein
Source: MedComm (2020). 2023 Jul 12;4(4):e319. doi: 10.1002/mco2.319 (PMC10338853; doi:10.1002/mco2.319)
Supplement: Supplementary file 1 — Supporting Information [file MCO2-4-e319-s001.docx]

Supplemental Materials

Matrix stiffness induced α-tubulin acetylation is required for skin fibrosis formation through activation of YAP

Dongsheng Wen^1#^, Ya Gao^1#^, Yangdan Liu^1#^, Chiakang Ho^1^, Jiaming Sun^1^, Lu Huang^1^, Yuxin Liu^1^, Qingfeng Li^1^*, Yifan Zhang^1^*

^1^Department of Plastic & Reconstructive Surgery, Shanghai Ninth People’s Hospital, Shanghai Jiao Tong University School of Medicine, Shanghai, China.

^#^These authors contributed equally to this work.

*Corresponding authors: Yifan Zhang and Qingfeng Li.

Address: 639 Zhizaoju Road, Depart of Plastic & Reconstructive Surgery, Shanghai Ninth People’s Hospital, Shanghai Jiao Tong University School of Medicine, Shanghai 200011, China.

E-mail addresses: zhangyifan82@126.com (Yifan Zhang), dr.liqingfeng@shsmu.edu.cn (Qingfeng Li).

This file includes:

Tables S1

Table S1. Volunteers’ information

| Volunteer | Sex | Age (years) | Localization | Time after trauma or burn (months) |
| --- | --- | --- | --- | --- |
| 1 | Female | 25 | Shoulder | 6 |
| 2 | Male | 33 | Chest | 10 |
| 3 | Female | 30 | Chest | 12 |
| 4 | Female | 38 | Back | 7 |
| 5 | Male | 45 | Chest | 6 |
| 6 | Male | 42 | Shoulder | 9 |
| 7 | Female | 19 | Chest | 7 |
| 8 | Male | 27 | Shoulder | 8 |
| 9 | Female | 28 | Trunk | 12 |
| 10 | Male | 24 | Back | 10 |
